# In Silico Screening of a Bile Acid Micelle Disruption Peptide for Oral Consumptions from Edible Peptide Database

**DOI:** 10.3390/foods10102496

**Published:** 2021-10-18

**Authors:** Kento Imai, Yuri Takeuchi, Kazunori Shimizu, Hiroyuki Honda

**Affiliations:** 1 Department of Biomolecular Engineering, Graduate School of Engineering, Nagoya University, Nagoya 464-8603, Japan; kento.keitai.171027@gmail.com (K.I.); takeuchi.yuri@f.mbox.nagoya-u.ac.jp (Y.T.); shimizu@chembio.nagoya-u.ac.jp (K.S.); 2 Japan Society for the Promotion of Science, Chiyoda-ku, Tokyo 102-0083, Japan

**Keywords:** edible protein, peptide database, peptide screening, oral administration, porous silica gel, cholesterol micelle

## Abstract

Recently, many bioactive peptides have been identified using bioinformatics tools. Previously, our group developed a method to screen dual-functional peptides that have direct intestinal delivery with porous silica gel and bile acid micelle disruption. However, newly designed peptides were not found in any storage protein. Therefore, in this study, in silico screening was performed using a 350,000 edible peptide library consisting of 4- to 7-mer independent peptides. As an initial screening, all edible peptides were applied to the random forest model to select predicted positive peptides. For a second screening, the peptides were assessed for the possibility of intestinal delivery using a 3D color map. From this approach, three novel dual-functional peptides, VYVFDE, WEFIDF, and VEEFYC were identified, and all of them were derived from storage proteins (legumin, myosin, and 11S globulin). In particular, VEEFYCS, in which a serine residue (S) is added to VEEFYC, was assumed to be released by thermolysin from the 11S-globulin derived from *Ginkgo biloba* by LC-MS/MS analysis. VEEFYCS was found to have suitable direct intestinal delivery and bile acid micelle disruption activity.

## 1. Introduction

Bioactive peptides provide health benefits to consumers, and many bioactive peptides have been identified from protein sources such as milk, plant seed, and seafood, among others [1]. The basic approach to identify bioactive peptides proceeds as follows: protein extraction, isolation, enzymatic hydrolysis, peptide purification, and verification of bioactivity in chemically synthesized peptides [1,2]. This “classical” method is laborious and time-consuming due to the trial-and-error process [3]. Recently, after identifying the peptides, most of the bioactive peptides have been determined from a database. One of the well-known databases is BIOPEP-UWM available at http://www.uwm.edu.pl/biochemia/index.php/en/biopep (accessed on 1 July 2021), which contains more than 4300 bioactive peptides and 740 proteins [4].

Recently, an integrated approach incorporating bioinformatics tools and in silico databases has been proposed for peptide identification and recovery toward commercialization [3,5]. Informatics tools help with screening by predicting bioactive peptides from the protein database. For instance, the binding affinity with target molecules is calculated by bioinformatics using the physicochemical properties of peptides. In a recent study, to select the most effective method to identify and recover dipeptidyl peptidase-4 (EC 3.4.14.5) inhibiting peptides from mealworm (*Tenebrio molitor*), Uniprot and BIOPEP enzyme action tools, ExPASy, were used [6]. In order to identify and recover the antioxidant peptides from flaxseed proteins effectively, informatics tools such as ExPASy and the ‘Peptides’ package in R were used [7]. In our previous study, α-amylase and α-glucosidase dual inhibitory peptides were screened by the random forests (RF) model using one amino acid-substituted peptide library based on GHWYYRCW [8]. Some bile acid-binding peptides have been screened by the principal component analysis (PCA) model from a randomly designed peptide library, and ExPASy was utilized to identify the peptides which could be isolated by enzyme hydrolysis [9,10]. Furthermore, bile acid-binding peptides from edible proteins were also screened using the RF model and a peptide database created from BIOPEP-UWM [11]. Bile acid-binding peptides can act as suppressors of cholesterol absorption via disruption of bile acid micelles including cholesterol molecules, resulting in a lowering of cholesterol levels in blood. Using this integrated approach, many bioactive peptides that could be isolated by enzyme hydrolysis have been identified within a short time.

However, many factors deter the industrial application of bioactive peptides. One of the biggest challenges is enzymatic degradation. Orally ingested peptides are inactivated by the action of digestive enzymes, such as peptidases and proteases in the stomach [12]. To overcome this problem, a heat-treated porous silica gel (HT silica gel) was developed that has an average pore size of 10 nm and a hydrophobic surface that changes depending on the environmental pH [13]. Using the HT silica gel, approximately 60% of the peptides located inside and/or on the surface of the HT silica gel were protected from pepsin proteolysis at pH 2.0. Consequently, it was concluded that the HT silica gel preferably desorbed hydrophobic and negatively charged peptides at pH 7 (intestinal environment), and the peptides with such properties enabled direct delivery to the intestinal space. To evaluate the intestinal delivery activity of any peptide, a 3D color map was designed, which consists of the physicochemical properties of peptides and intestinal delivery score [13]. In a subsequent study, dual-functional peptides were developed [14], which have properties for direct intestinal delivery and bile acid-binding activity, utilizing the analysis of residue-substituted peptides of parent peptide, VAWWMY [15]. As a result, two novel peptides, IYWEMY and IYEYMY, were obtained.

VAWWMY was released from the glycinin A1aB1b subunit of the soybean protein However, the newly designed peptides, IYWEMY and IYEYMY, have not been found in any storage proteins to the best of our knowledge. Therefore, the screening method developed previously needed to be improved.

In the development of bioactive peptides for human health, toxic proteins such as cytolytic protein, neurotoxin and enterotoxin, are unable to be utilized. Traditional proteins known as safe food sources are preferable for human consumption. In our previous study, a peptide database was constructed from BIOPEP-UWM, which is called an edible peptide database [11]. This database consists of peptide fragments derived from food proteins that have one residue shift from the N-terminal amino acid. The peptides within database have been found in nature. 

Here, in silico screening of dual-functional peptides was performed from storage proteins, bile acid micelle disruption activity, and intestinal delivery using an HT silica gel. Some novel peptides were screened from storage proteins using this approach. The identified peptide showed high activity for both micelle disruption and direct intestinal delivery.

## 2. Materials and Methods

### 2.1. Materials

The following Fmoc-protected amino acids were purchased from Watanabe Chemical Industries, Ltd. (Hiroshima, Japan): Fmoc-Asp(OtBu)-OH, Fmoc-Glu(OtBu)-OH, Fmoc-Ile-OH, Fmoc-Leu-OH, Fmoc-Phe-OH, Fmoc-Thr(tBu)-OH, Fmoc-Val-OH, Fmoc-Cys(Acm)-OH, Fmoc-Tyr(OtBu)-OH, Boc-Cys(Trt)-OH. HT silica gel, SMB-100-5 was supplied by Fuji Silysia Chemical Ltd. (Kasugai, Aichi, Japan). Cholesterol (038-03005), oleic acid (159-00246), cholesterol kit (439-17501), fluorescamine (061-03831), and hexane (085-00416) were purchased from Fujifilm Wako Pure Chemical Corporation (Osaka, Japan). Monoolein (23408-12) and phosphatidylcholine from egg yolk (27554-01) were purchased from Nacalai Tesque (Kyoto, Japan). Taurocholic acid sodium salt hydrate (T-4009) and thermolysin (33607-34) were purchased from Sigma-Aldrich (St. Lousis, MO, USA). EDTA (0.5 mM pH 8.0, 15575020) was purchased from Thermo Fisher Scientific (Waltham, MA, USA).

### 2.2. First Screening

**Figure 1:**
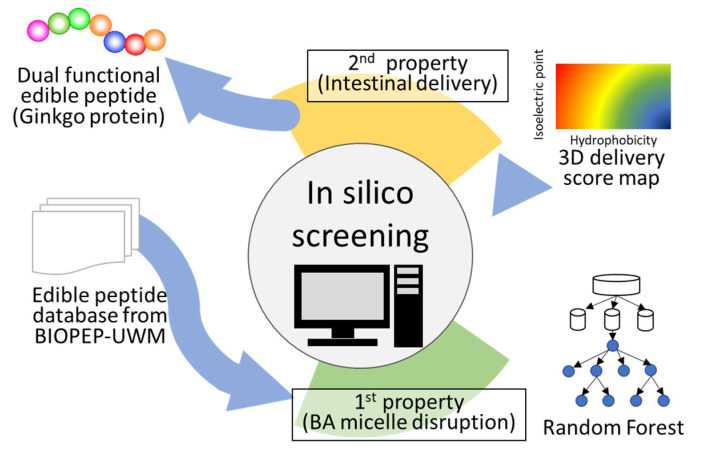
Schematic of the method for peptide screening. A peptide database created from BIOPEP-UWM was used as a peptide library. The peptides stored in the library were adapted to the random forest model. Peptides with scores of >0.5, predicted to be positive, were used for further screening. All candidate peptides were plotted on the color map, and peptides with scores >50 were selected and subjected to in vitro assays. Five peptides were selected using this method.

The flow of the in silico screening is shown in Figure 1. The peptide database created previously from the protein database, BIOPEP-UWM, available at http://www.uwm.edu.pl/biochemia/index.php/en/biopep (accessed on 23 October 2018). was used for screening peptides [11]. On the date we accessed it (23 October 2018), 710 protein sequences were stored in the database. A set of 710 proteins was downloaded from BIOPEP-UWM, and sequences were divided into peptide fragments with one residue shift from the N-terminal, resulting in a peptide database. First, the peptides stored in the database were adapted to the RF model created previously [11] and those with scores > 0.5, which are predicted as positive because the probability was a range from 0 to 1, were used for further screening.

### 2.3. Second Screening

A color map was proposed by us as an indicator of HT silica gel delivery [13,14]. Here, 4-, 5-, 6-, and 7-mer color maps were drawn as in our previous study. Peptides that passed through the first screening were plotted on the color map after the coordinates of all peptides were defined as follows: 

Two indices, hydrophobicity [16] and isoelectric point (pI) [17], were chosen as the axis of the graph, because those are related to hydrophobic and electrostatic interactions with biomolecules. The properties of peptides were calculated based on the following equations: For example, in the case of 4-mer peptides,

Hydrophobicity = (Xi1 + Xi2 + Xi3 + Xi4)/4(1)

pI = (Yi1 + Yi2 + Yi3 + Yi4)/4(2)

where Xi and Yi are the hydrophobicity and pI values of the amino acids in the peptide, respectively. Subscripts 1, 2, and 3 indicate the first, second, and third amino acids from the N-terminal of peptide, respectively. Thus, Xi and Yi indicate the average hydrophobicity and pI of the peptide, respectively. A 3D color map was created using MATLAB. The z-axis is the score on the HT silica gel delivery property [13]. All peptides that had passed the first screening were plotted on the color map, and peptides with a high score were selected as candidate peptides for in vitro assays. The threshold value of 50 was chosen to select less than 10 candidate peptides.

### 2.4. Assessment of Peptide Release under pH 7 Condition

Peptide adsorption and desorption experiments were conducted as described previously [13,14]. A silica suspension of 150 μL (25 mg/mL) and 150 μL of peptide solution (0.5 mM) were prepared and shaken with a vortex mixer for 30 s and left to equilibrate for 5 min at 25 °C. After centrifugation at 9300× *g* for 1 min to remove the supernatant, 300 μL of phosphate buffer (pH 2.1) was added to the silica gel and shaken vigorously, and left to equilibrate for 5 min at room temperature to release the peptide under acidic conditions. Next, the mixture was separated in the same way and 300 μL of PBS (pH 7.4) was added to the silica gel, shaken vigorously, and left to equilibrate for 5 min at room temperature to release the peptide under neutral pH conditions. The peptides released were quantified using a fluorimetric assay. Ten microliters of fluorescamine (5 mg/mL in acetone) were added to a 150 μL aliquot of the supernatant in a 96-well plate, and the fluorescence intensity was measured with excitation at 355 nm and emission at 460 nm (Fluoroskan Ascent Microplate; Thermo Fisher Scientific, USA).

### 2.5. Peptide Synthesis

Identified peptides were synthesized on Fmoc-Ile-Alko Resin (K00533, Watanabe Chemical Industries, Ltd.), Fmoc-Val-Alko Resin (K00545, Watanabe Chemical Industries, Ltd.), Fmoc-Trp-Alko Resin (K00543, Watanabe Chemical Industries, Ltd.), Fmoc-Glu(OtBu)-Alko Resin (K00530, Watanabe Chemical Industries, Ltd.) using Polypropylene columns (2600030, KOKUSAN Chemical Co. Ltd., Tokyo, Japan). This method was based on the standard Fmoc solid-phase peptide synthesis method, as described below. Briefly, the coupling reaction was terminated with 2-(1H-Benzotriazole-1-yl)-1,1,3,3-tetramethyluronium hexafluorophosphate (A00149, Watanabe Chemical Industries, Ltd.) and *N*-methylmorpholine (19-3775-5, Sigma-Aldrich Japan, Tokyo, Japan). The Fmoc group was removed by 20% piperidine (A00177, Watanabe Chemical Industries, Ltd.) in *N*,*N*-dimethylformamide (10344-80, Kanto Chemical Co., Inc., Tokyo, Japan). Then, 20 mL of depolymerization cocktail (81.5% trifluoroacetic acid (A00026, Watanabe Chemical Industries, Ltd.), 10% distilled water, 5% thioanisole (T0191, Tokyo Chemical Industry Co., Ltd., Tokyo, Japan), 2.5% 1,2-ethylene glycol (E0032, Tokyo Chemical Industry Co., Ltd.), and 1% triisopropylsilane (A00170, Watanabe Chemical Industries, Ltd.) were added to the resin in which the peptide was synthesized and shaken overnight. Following this, 40 mL diethyl ether (14134-81, Kanto Chemical Co., Inc.) was added and centrifuged at 3000× *g* at 0 °C for 10 min. The supernatant was removed and washed three times with tert-butyl ether. Finally, precipitates were lyophilized, and peptides were stored as dried samples.

### 2.6. Bile Acid Micelle Preparation and the Determination of the Disruption Activities

The bile acid disruption assay was performed according to a previous study [14]. Bile acid (BA) micelles were prepared as described in Raederstorff et al. [18] and Nagaoka et al. [19]. The mixture of 0.5 mmol/L cholesterol, 1 mmol/L oleic acid, 0.5 mmol/L monoolein, and 0.6 mmol/L phosphatidylcholines was prepared in methanol and dried for 24 h. Phosphate buffer saline (PBS) containing 6.6 mmol/L sodium taurocholate was added and micelles were formed by sonication for 30 min. After incubation at 37 °C for 24 h, candidate peptides (synthesized using Fmoc-based synthesis) were added to the micellar solutions. After incubation at 37 °C for 1 h, the solution was centrifuged at 10,000 × g for 20 min to separate the precipitated cholesterol, and the supernatant was filtered through a 0.22 µm filter, and filtrate (0.5 mL) was added to 3 mL of the cholesterol kit solution. The absorbance was measured at 600 nm. The concentration of cholesterol in the BA micelles was calculated using a calibration curve. Dose dependency was fitted by a sigmoid curve using an R script written by us with R software (version 3.5.3, Murray Hill, NJ, USA) (R development Core Team, https://www.r-project.org/ (accessed on 13 October 2020)), resulted in DC50 (50% cholesterol concentration decrease value, defined previously [9]) 

### 2.7. Cleavage Site Prediction

ExPASy: PeptideCutter (https://web.expasy.org/peptide_cutter/ (accessed on 13 October 2020)), which is one of the important tools for bioactive peptide screening [7], was used for cleavage site prediction, and all enzymes listed on this site were used as simulation enzymes.

### 2.8. Preparation of Ginkgo Protein from Defatted Ginkgo Flour

Ginkgo (*Ginkgo biloba*) is one of the famous street trees in Japan. Ginkgo nuts are also a familiar food and frequently used in Japanese cuisine. Ginkgo nuts were collected at the Higashiyama campus of Nagoya University (Japan) and peeled to obtain the seeds. Ginkgo seeds were defatted according to the method described in Liu et al. (2007) [20]. Briefly, the seeds were freeze-dried and milled. Ginkgo flour was defatted by hexane extraction (Ginkgo flour/hexane = 1:5, *v*/*v*) for 1 h at room temperature. After centrifugation (8000× *g*, 15 min, 4 °C), the supernatant was discarded and the precipitate was extracted twice. The defatted flour was then dried overnight at room temperature. 

Ginkgo protein was extracted according to the method described in Deng et al. (2011) [21]. The defatted ginkgo flour was dissolved in ultra-pure water (ginkgo flour/water = 1:9 *v*/*v*) and stirred for 10 min. Then, NaOH was added to the flour solution until the pH reached 9.0 and stirred for 30 min. After centrifugation (4000× *g*, 4 °C, 15 min), the supernatant was collected. The precipitate was extracted twice. The pH of the supernatant was adjusted to 4.4 with HCl and kept for 30 min at room temperature. After centrifugation (4000× *g*, 4 °C, 20 min), the supernatant was discarded. The precipitate was washed twice with water and then freeze-dried.

### 2.9. Hydrolysis of Ginkgo Protein

Ginkgo protein was dissolved in 50 mM Tris-HCl including 0.5 mM CaCl_2_ (pH = 7.0) and thermolysin was dissolved in 50 mM Tris-HCl (pH 7.0). The protein and thermolysin solutions were mixed (protein /thermolysin = 15:1 *v*/*v*) and incubated for 30 min at 50 °C. Hydrolysis was stopped by adding 0.5 mM EDTA (pH 8.0) (mixture/EDTA = 10:1). After centrifugation (8000× *g*, 4 °C, 10 min), the supernatant was freeze dried.

Protein profiles of the ginkgo protein and its hydrolysates were analyzed by sodium dodecyl sulfate-polyacrylamide gel electrophoresis (SDS-PAGE) [22]. 

### 2.10. LC-MS Analysis to Identify Peptides in the Hydrolysate 

Samples were analyzed by nano-flow reverse-phase liquid chromatography followed by tandem MS. A capillary reverse-phase HPLC-MS/MS system (Thermo Fisher Scientific, USA) composed of a Dionex U3000 gradient pump equipped with a VICI CHEMINERT valve, and Q Exactive equipped with a nano-electrospray ionization (NSI) source (AMR, Japan) was used. The desalted peptides were trapped on a 5 mm × 100 µm ID trap column packed with 5 µm 120 C18 resin and separated at 500 nL/min using a 5–40% buffer B gradient over 100 min on a NANO-HPLC capillary column C18 (0.1 × 125 mm, Nikkyo Technos). The composition of the LC buffer A was 0.5% (*v*/*v*) acetic acid in water and LC buffer B was of 80% (*v*/*v*) acetonitrile, 0.5% (*v*/*v*) acetic acid. The Xcalibur 3.0.63 system (Thermo) was used to record peptide spectra over the mass range of *m*/*z* 350–1800 (70,000 resolution, 3e6 AGC, 60 ms injection time). MS spectra were recorded, followed by ten data-dependent high-energy collisional dissociations (HCD) MS/MS spectra generated from ten highest-intensity precursor ions (17,500 resolution, 1e5 AGC, 60 ms injection time, 27 NCE). MS/MS spectra were interpreted and peak lists were generated using Proteome Discoverer 2.4.1.15 (Thermo). Searches were performed using SEQUEST (Thermo) against the Ginkgo protein database. Peptide identifications were based on a significant Xcorr (high-confidence filter). Peptide identification and modification information determined from SEQUEST were manually inspected and filtered to obtain confirmed peptide identification and modification lists of HCD MS/MS. The eluents used were (A) 100% water containing 0.5% acetic acid, and (B) 80% acetonitrile containing 0.5% acetic acid. The column was developed at a flow rate of 0.5 μL/min with the concentration gradient of acetonitrile: from 5% (B) to 40% (B) in 20 min, 40% B to 95% (B)in 1 min, sustaining 95% (B) for 3 min, from 95% (B) to 5% (B) in 1 min, and finally re-equilibrating with 5% (B) for 10 min.

### 2.11. Statistical Analysis

Each experiment was performed in triplicate. The means and standard deviations (SDs) were calculated and statistical analysis was performed by Student’s *t*-test.

## 3. Results

### 3.1. Peptide Classification by the RF Model

A set of 710 edible proteins was downloaded from BIOPEP-UWM, and sequences were divided into 4-mer, 5-mer, 6-mer, or 7-mer peptide fragments with one residue shift from the N-terminal, resulting in up to 350,000 independent peptides. All peptide sequences were applied to the RF model developed previously [11]. The peptides were classified as positive or negative according to their predicted bile acid-binding activity (Table S1). 

### 3.2. Prediction of Delivery Properties Using Color Maps

**Figure 2:**
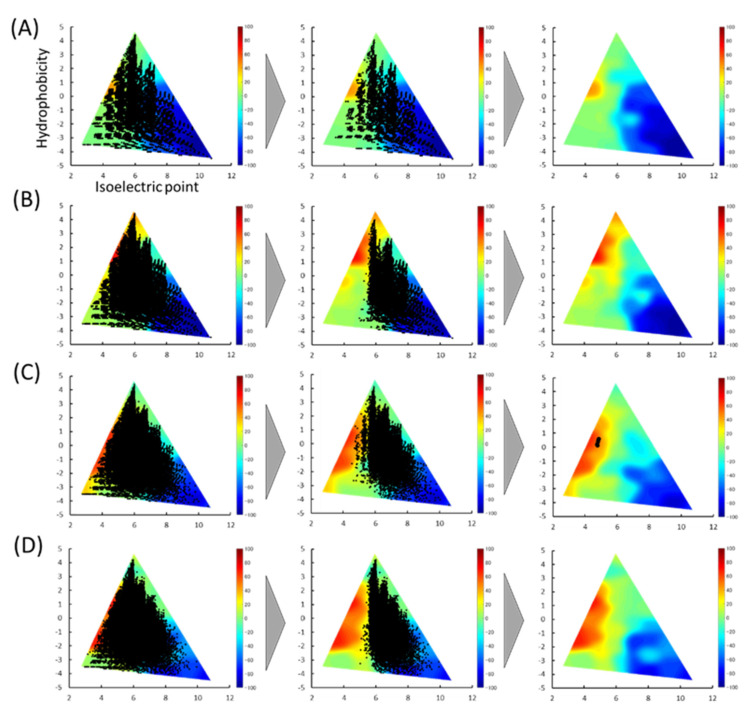
In silico prediction of delivery properties with porous silica gel and selected peptides. Color maps classified by hydrophobicity versus isoelectric point (pI) based on 4-mer (**A**), 5-mer (**B**), 6-mer (**C**), 7-mer (**D**). In the left columns, all peptides stored in the library are indicated as black circles on the color map. In the middle columns, all peptides predicted to be positive are plotted as black circles. In the right columns, peptides with scores >50 are denoted as black circles.

We reported a 3D color map based on the physicochemical properties of the peptides to estimate the intestinal delivery properties using HT porous silica gel [13]. The pI and hydrophobicity of the peptides classified as positive were calculated and plotted on color maps (Figure 2). The higher the score, the more suitable the characteristic peptide. As shown in the left columns, all peptide-stored databases were scattered throughout the map. After the first screening, the peptides were biased from the center to the right sides of the map. The average distribution of peptides on the 3D color map was investigated before and after the first screening. While the average pIs of all peptides stored in the database were almost 6.0, those after the first screening were found to be shifted from 0.6 to 0.7 (Table 1). 

Next, a second screening by delivery score was performed. Peptides with a score >50 were selected, resulting in only five peptides being screened. The peptides were all with six residues: VYVFDE, IFIYDE, WEFIDF, VEEFYC, and ELYEFC. Details of the parent protein of the five peptides are shown in Table 2. 

Since they were obtained from storage proteins, the peptides had high natural content and are very promising for industrial applications. Next, we synthesized the peptides and evaluated their bioactivity and delivery properties in vitro.

### 3.3. Evaluation of Micelle Disruption Activity

**Figure 3:**
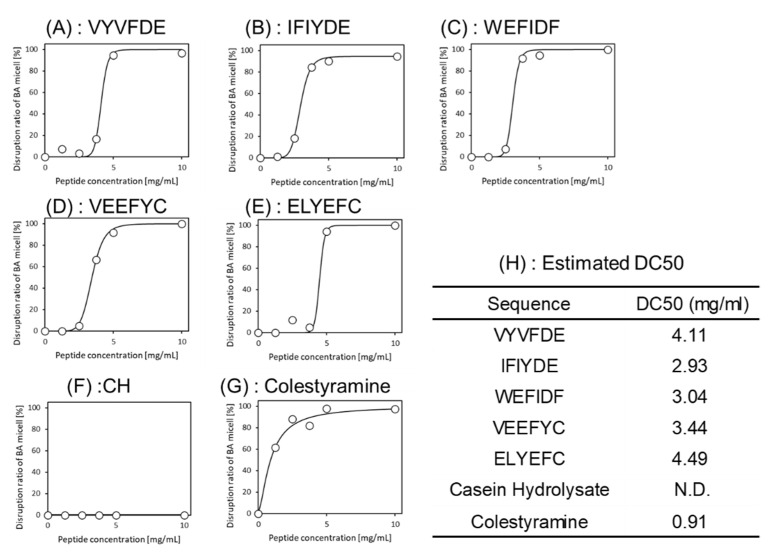
Dose-response curves of the peptides for micelle disruption activity. (**A**) VYVFDE, (**B**) IFIYDE, (**C**) WEFIDF, (**D**) VEEFYC, (**E**) ELYEFC, (**F**) Casein hydrolysates (negative control), (**G**) Colestyramine (positive control). (**H**) 50% cholesterol concentration decrease values (DC50s) as with EC50 were estimated by sigmoid curve fitting with R software.

The above two-step screening was conducted in silico. The bioactivity of five peptides identified was assured by actual assay. Bioactivity was evaluated by adding the synthesized peptides to a BA micelle solution. All peptides showed 100% disruption activity at concentrations above 5 mg/mL. To investigate the dose-dependency of the five peptides, the same experiments were conducted with varying concentrations and fitted to a sigmoid curve. As shown in Figure 3, the estimated DC50s were 4.11 mg/mL (VYVFDE), 2.93 mg/mL (IFIYDE), 3.04 mg/mL (WEFIDF) and 3.44 mg/mL (VEEFYC) (Figure 3H). The activity of the ELYEFC was slightly lower. Although these scores cannot be compared to cholestyramine, which was used as a positive control, four selected peptides showed high micelle disruption activities.

### 3.4. Evaluation of the Release Amount from HT Silica Gel

**Figure 4:**
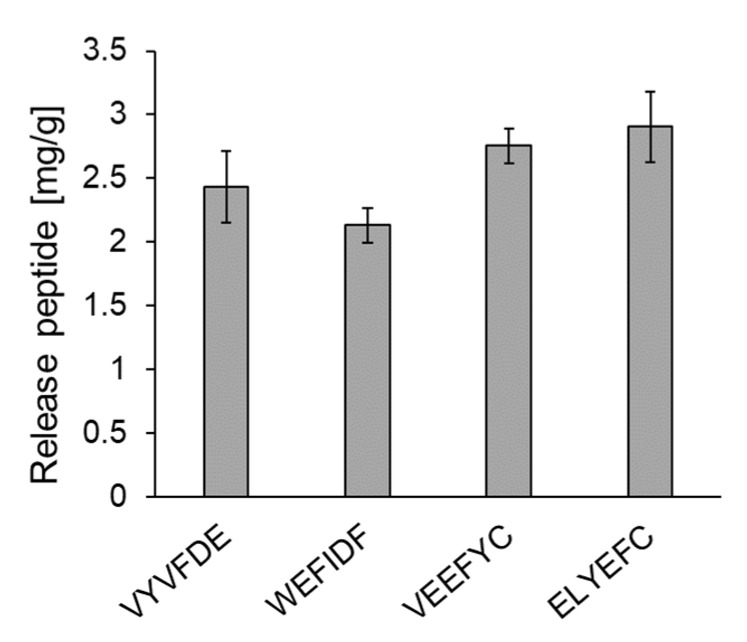
Amount of the five peptides released at pH 7. First all peptides were adsorbed to HT silica gel at pH 2. IFIYDE could not tested because it did not dissolve under at pH 2.

The release amounts of five peptides from HT silica gel were evaluated. Since we reported two micelle disruption peptides, IYEYMY and IYWEMY, in our previous study [14], the intestinal delivery of the five peptides were compared with those such as 2.96 mg/g for IYEYMY and 2.30 mg/g for IYWEMY. As shown in Figure 4, the following release amounts were obtained: VYVFDE (2.43 ± 0.28 mg/g), WEFIDF (2.13 ± 0.14 mg/g) and VEEFYC (2.76 ± 0.14 mg/g). IFIYDE could not be tested since it did not dissolve under pH 2.0 conditions.

**Figure 5:**
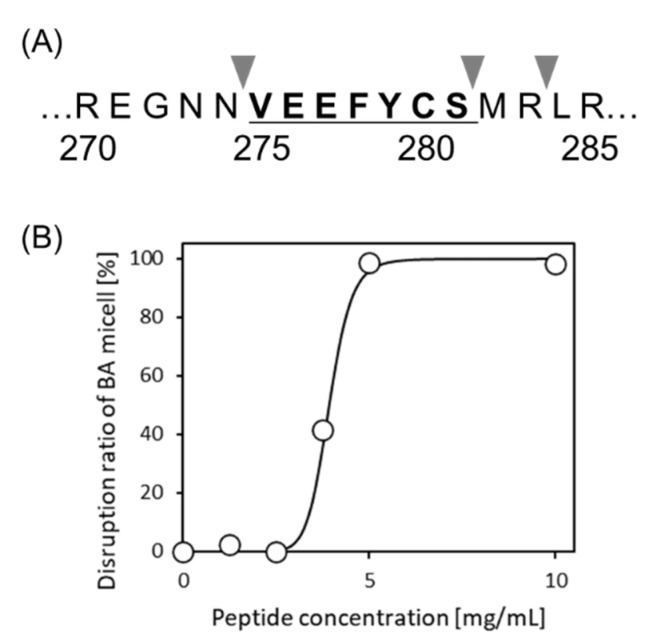
Cleavage of VEEFYCS in 11S-globulin and micelle disruption activity. (**A**) Cleavage sites for thermolysin on 11S-globulin (Q39770) predicted with PeptideCutter. (**B**) Dose-response curve of VEEFYCS in micelle disruption activity.

It is still unknown whether VYVFDE, WEFIDF, and VEEFYC could be released by proteases. We used PeptideCutter available at https://web.expasy.org/peptide_cutter/ (accessed on 30 September 2020) to predict the release of these peptides via protein hydrolysis. It was seen that two of them could not be released intact; however, in the case of VEEFYC, this could be cleaved from 11S-globulin (Q39770) when thermolysin was used as the cleavage enzyme (Figure 5A).

### 3.5. Separation of VEEFYCS from Ginkgo Protein

Alkaline protein extraction was conducted from ginkgo nuts and enzyme hydrolysis of ginkgo protein. The molecular weight of legumin, i.e., ginkgo 11S-globulin, was 51.450 kDa (Q39770, Uniprot). Molecular weight after removed the signal peptide (1–25) was 48.637 kDa. It was suggested that the extracted ginkgo protein included legumin, since a band at 50 kDa was observed. Furthermore, it was also indicated that the ginkgo protein was sufficiently hydrolyzed by thermolysin (Figure S1). 

The hydrolysates and synthesized VEEFYCS peptides were analyzed by LS-MS/MS. For detecting various peptides including VEEFYCS from the hydrolysate, nontargeted analysis was conducted. As shown in Figure S2A, mass spectrometry of the VEEFYCS peptide showed a single peak with an *m*/*z* of 876.3480. Signals at 876.3480 and 438.6773 were [M + H]^+^, [M + 2H]^2+^, respectively. Since mass spectrometry of the hydrolysates revealed a peak with the same *m*/*z* value (Figure S2B), it was suggested that VEEFYCS could be obtained by hydrolyzing the ginkgo protein with thermolysin for 30 min at 50 °C. 

As a preliminarily experiment, no pretreatment of extracted protein, such as the reduction of the probable disulfide bonds, or an alkylation, was conducted. Here, although the cysteine residue was included in sequence, naïve VEEFYCS was detected by LC-MS/MS. Häger et al. [23] reported that ginkgo legumin is divided into α- and β-subunit by posttranslational cleavage and the cysteine residue of VEEFYCS is most likely to be forming the disulfide bridge linking between two subunits from the sequence homology with other angiosperm legumins such as *Oryza sativa*, *Pisum sativum* and *Glycine max*. Although the involvement of the interchain disulfide linkage was expected, it was considerable that VEEFYCS was released from free subunit before association by thermolysin reaction since VEEFYCS is located in adjacent to the cleavage site. 

### 3.6. Bioactivity of VEEFYCS

A newly synthesized 7-mer peptide VEEFYCS was investigated for micelle disruption activity and its release from the HT silica gel. A suitable disruption activity was obtained as shown in Figure 5B. The DC50 was 3.9 mg/mL. The release amount from HT silica gel was 1.5 mg/g. From this prediction, VEEFYCS was found to be a promising candidate, even if a serine residue (S) is added to VEEFYC.

## 4. Discussion

In this study, a screening method was proposed for exploring dual-functional peptides derived from storage proteins based on machine learning. The functions analyzed included BA micelle disruption activity for cholesterol reduction and direct intestinal delivery without hydrolysis using HT silica gel. Consequently, three novel peptides, VYVFDE, WEFIDF, and VEEFYC, were screened using a peptide database, and VEEFYCS was predicted to be released from the 11S-globulin (Q39770) with thermolysin. Moreover, VEEFYCS was released from the hydrolysate of Ginkgo protein with thermolysin. From 1g of ginkgo nuts, 0.216 g of 11S ginkgo protein was obtained by our purification. The 11S legumin is one of the major seed storage proteins. Assuming that 15.7% of alkaline extracted protein is 11S-globulin (legumin) from the image analysis SDS-PAGE in Figure S1, approximately 33.9 mg of legumin will be obtained from 1 g of nuts. At most, 0.52 mg (=33.9 × 7/460 mg) of the peptide will be finally purified, since Q39770 protein is 460 AAs.

First, more than 350,000 peptides were listed from a set of 710 edible proteins stored in BIOPEP-UWM. All peptides were classified by the RF model constructed previously, and a large number of peptides were predicted to be positive from the RF model. As shown in Table S1, the ratio of positive peptides was 34% for the 6-mer library, which is the lowest value, whereas that for the 7-mer library was 49%, which is the highest value. These degrees of the positive prediction ratio were reasonable because 150 peptides (33%), among 460 total random peptides were selected as positive learning data in the RF model [11].

All peptide-stored databases were scattered throughout the 3D color map for intestinal delivery. After the first screening, the peptides were biased from the center to the right sides of the map. This is because positively charged peptides are more likely to be screened since bile acids are negatively charged. Previous research has revealed that hydrophobic, especially aromatic, and basic groups interact with BA micelles [9,10,24]. The RF model utilized here also selected similar features relating to isoelectric points and molecular weights [11]. Therefore, this consideration could explain the biased plots after the first screening. A similar tendency was observed in our previous PCA analysis [10]. 

Five peptides were selected by an in silico two-step screening. The bioactivity of these peptides were assured by actual assays. The BA micelle disruption activities of the five peptides were evaluated. Surprisingly, all peptides showed 100% disruption activity at concentrations above 5 mg/mL as shown in Figure 3. The release amounts of the five peptides were also evaluated. As shown in Figure 4, the release amount of each peptide was 2–3 mg/g. These values are relatively high. The reason why five peptides show high activities and high release amounts could be explained using amino acid content of these five peptides as the follows.

**Figure 6:**
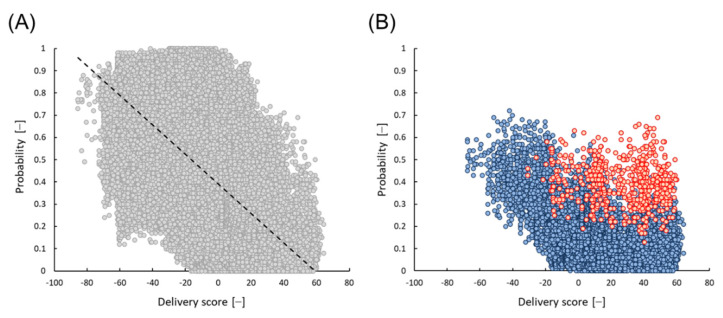
Relationship between the probability of RF model and the predicted delivery score on the 6-mer edible peptides derived from storage proteins. (**A**) Total 98,387 peptides (gray dots, a dashed line means a least square approximate straight line), (**B**) 15,609 peptides containing more than two residues of acidic amino acid such as D (Asp) or E (Glu) (blue dots), and 589 peptides additionally containing more than two residues of aromatic amino acids such as F (Phe), Y (Tyr), or W (Trp) (red dots).

Amino acid (AA) distributions of 98,387 peptides from the 6-mer library were analyzed. As shown in Table 3, a larger number of positives were obtained when basic AAs were present and acidic AAs were not. The odds ratios were 6.5 and 22, respectively. This tendency was also confirmed for the AA distribution of 150 positives using the RF model (Table S2). The relationship between the probability of the RF model and the delivery score of 6-mer 98,387 peptides is shown in Figure 6A, and there was an opposite correlation. All five peptides selected in this study contained two acidic AAs. All 15,609 peptides, which contain more than two acidic AAs were mostly plotted in areas with higher delivery scores and lower probabilities (blue symbols in Figure 6B). However, even though acidic AAs were contained in a peptide, it was found that the peptide showed relatively high micelle disruption activity if aromatic AAs were also present. For instance, all 589 peptides that contain more than two aromatic AAs showed a relatively high probability (red symbols in Figure 6B). The average probabilities of 15,609 and 589 peptides were 0.15 and 0.40, respectively. This bias was also observed in the AA distributions listed in Table 3 and Table S2. Since all five peptides selected in the present study contained two acidic AAs and more than two aromatic AAs, it was likely that the peptides caused high micelle disruption activity. 

Five peptides were extracted after the second screening. As shown in Table 2, VYVFDE is derived from legumin (globulin-like protein) contained in *Zea maize* (Q946V2; UniProt accession number). WEFIDF is derived from myosin in *Gallus* and common carp (P13538 and Q90339, respectively). WEFIDF was searched in detail with UniProt and it was found that WEFIDF is a widely conserved sequence in myosin, and this sequence could be found in a wide range of animals, such as *Sus scrofa* (P79293), *Oryctolagus cuniculus* (Q28641), *Gallus*, and common carp. VEEFYC is derived from 11S globulin in *Ginkgo biloba* (Q39770). All origins were quite different from each other. It was confirmed that a bioactive peptide could first exert its activity after proteolytic release and is not reflected by protein properties. 

## 5. Conclusions

In conclusion, three novel peptides, VYVFDE, WEFIDF, and VEEFYC, were identified, which were all derived from storage proteins (legumin, myosin, and 11S-globulin, respectively) using an edible peptide database. After PeptideCutter analysis, the 7-mer peptide VEEFYCS was selected (Figure 5A). It was found that VEEFYCS showed similar micelle disruption activity and similar release amount from HT silica gel compared with 6-mer peptide VEEFYC. In addition, VEEFYCS seems to be the most suitable for practical applications even if serine (S) is added to VEEFYC using thermolysin, since this peptide showed high activity for both micelle disruption and direct intestinal delivery. It was concluded that new BPs derived from edible proteins can be successfully identified using in silico screening proposed here without conventional techniques on separation, purification or recovery of peptides. 

## Figures and Tables

**Table 1:** Distribution average of peptides on a 3D color map before and after the first screening.

	Isoelectric Point		Hydrophobicity	
Library	Before	After	Before	After
4-mer	6.03 ± 0.90	6.63 ± 0.90	−0.46 ± 1.53	−0.83 ± 1.51
5-mer	6.01 ± 0.80	6.71 ± 0.65	−0.41 ± 1.43	−0.71 ± 1.33
6-mer	6.00 ± 0.73	6.61 ± 0.61	0.41 ± 1.34	−0.34 ± 1.28
7-mer	6.00 ± 0.68	6.63 ± 0.54	−0.42 ± 1.26	−0.30 ± 1.36

**Table 2:** The characteristics of candidate peptides. Protein: parent protein. Position: peptide position from the N-terminus. This number was determined using sequences stored in the BIOPEP-UWM. Some of them were different from those in UniProt.

Peptide	Protein	Position
VYVFDE	legumin 1, maize (*Zea maize*) *	185_190
IFIYDE	Na/K ATPase alpha subunit isoform 2, rainbow trout (*Oncorhynchus mykiss*)	987_992
WEFIDF	myosin subfragment-1, chicken (*Gallus gallus*) *	510_515
WEFIDF	Myosin heavy chain, *Cyprinus carpio* (Common carp) (Q90339) *	510_515
VEEFYC	11S-globulin (b-legumin-like chain), ginkgo (*Ginkgo biloba*) *	255_260
ELYEFC	G10 protein homolog, rice (*Oryza sativa*)	72_77

Abbreviations; C: Cys, D: Asp, E: Glu, F: Phe, I: Ile, V: Val, Y: Tyr, W: Trp. * Storage proteins.

**Table 3:** Amino acid (AA) distribution of positives and negatives predicted by RF modeling.

Category	Total	Positives	Negatives	Odds Ratio
with R, K	46,616	25,318	21,298	6.45
w/o R, K	51,771	8055	43,716	
with D, E	48,390	3135 (with F, Y, W; 1877)	45,255 (with F, Y, W; 14,682)	
w/o D, E	49,997	30,238	19,759	22.1
with F, Y, W	37,883	17,646	20,237	2.48
w/o F, Y, W	60,504	15,727	44,777	
Total	98,387	33,373	65,014	

Abbreviations; w/o: without, R: Arg, K: Lys, D: Asp, E: Glu, F: Phe, Y: Tyr, W: Trp.

## Data Availability

Data is contained within the article or Supplementary Materials.

## Supplementary Materials

The following are available online at www.mdpi.com/article/10.3390/foods10102496/s1. Figure S1: SDS-PAGE analysis of ginkgo protein. Figure S2: Mass spectra of (A) VEEFYCS and (B) ginkgo protein hydrolysate. Table S1: The number of peptide libraries and positive and negative peptides. Table S2: Amino acid (AA) distribution of 150 positives and 150 negatives from RF modeling.
