# Supplementary material for: In Silico Screening of a Bile Acid Micelle Disruption Peptide for Oral Consumptions from Edible Peptide Database
Source: Foods. 2021 Oct 18;10(10):2496. doi: 10.3390/foods10102496 (PMC8536064; doi:10.3390/foods10102496)
Supplement: Supplementary file 1 [file foods-10-02496-s001.zip › foods-1413830-supplementary.pdf]

In Silico Screening of a Bile Acid Micelle Disruption Peptide for Oral Consumption from Edible Peptide Database

Kento Imai [1,2], Yuri Takeuchi [1], Kazunori Shimizu [1] and Hiroyuki Honda [1,*]

[1] *Department of Biomolecular Engineering, Graduate School of Engineering, Nagoya University, Nagoya 464-8603, Japan; kento.keitai.171027@gmail.com (K.I.); takeuchi.yuri@f.mbox.nagoya-u.ac.jp (Y.T.); shimi-zu@chembio.nagoya-u.ac.jp (K.S.)*

[2] *Japan Society for the Promotion of Science, Research Fellowship for Young Scientists, Chiyoda-ku, Tokyo 102-0083, Japan*

* Correspondence: honda@chembio.nagoya-u.ac.jp

The supplementary information contains the following sections:

Figures S1 and S2, Tables S1 and S2

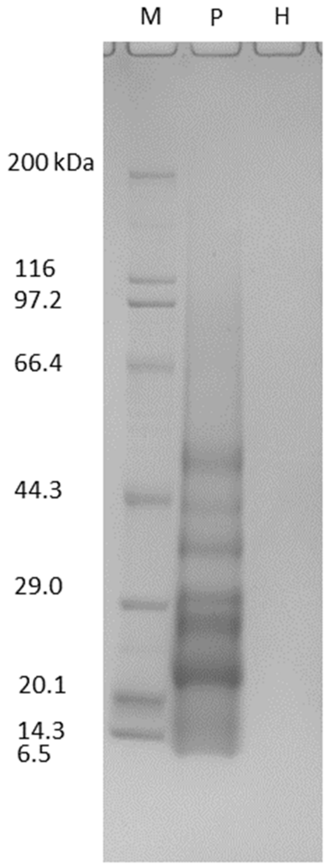

Figure S1

SDS-PAGE analysis of ginkgo protein. Ginkgo protein (lane P) and hydrolysate (lane H) samples were analyzed using a 5–20 % SDS gel. The molecular weights of the protein bands in Protein Molecular Weight Marker Broad (lane M; kDa) are shown to the left of the gel.

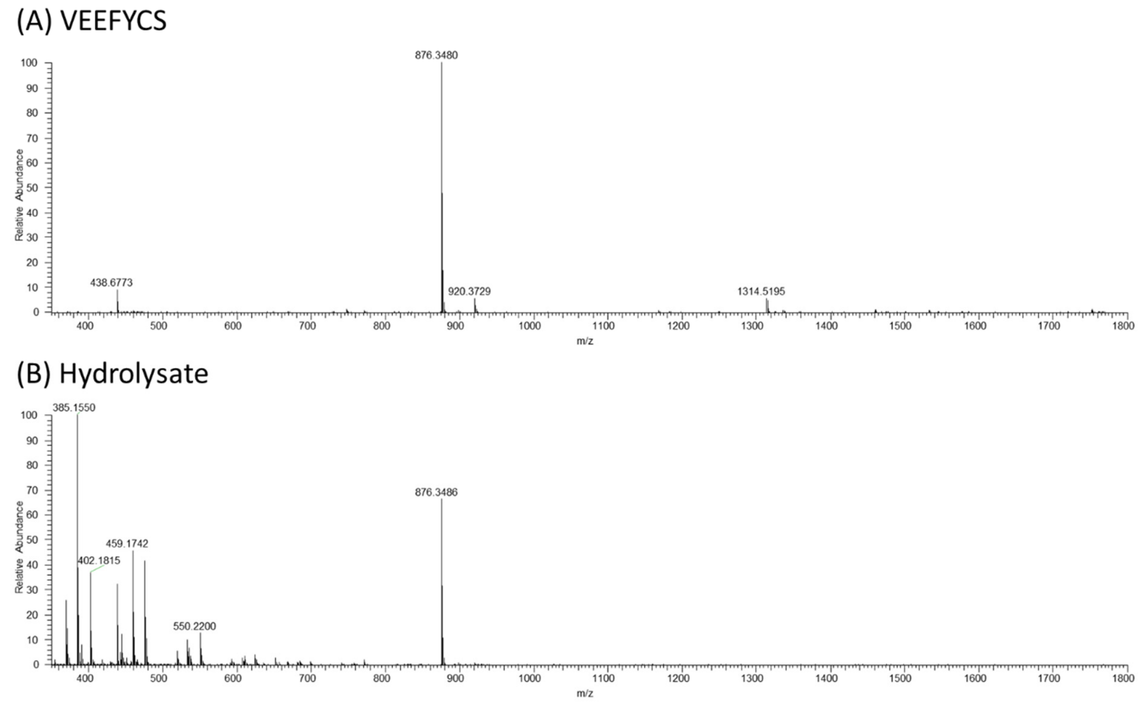

Figure S2

Mass spectra of (A) VEEFYCS and (B) ginkgo protein hydrolysate.

Table S1

The number of peptide libraries and positive and negative peptides

Residue	4	5	6	7
1 (Positive)	19667	35158	33373	50796
Positive ratio	35%	39%	34%	49%
0 (Negative)	36504	54505	65014	52009
Total	56171	89663	98387	102805

Table S2 Amino acid (AA) distribution of 150 positives and 150 negatives from RF modeling

Category	Total	Positives	Negatives	Odds ratio
with R, K	251	120	60	6.00
w/o R, K	209	30	90	
with D, E	234	21<br>(with F, Y, W; 19)	116<br>(with F, Y, W; 58)	
w/o D, E	226	129	34	21.0
with F, Y, W	323	124	74	4.90
w/o F, Y, W	137	26	76	
Total	460	150	150	

Abbreviations; w/o; without, R: Arg, K: Lys, D: Asp, E: Glu, F: Phe, Y: Tyr, W: Trp.
